# Global microRNA expression profiling: Curcumin (diferuloylmethane) alters oxidative stress-responsive microRNAs in human ARPE-19 cells

**Published:** 2013-03-15

**Authors:** Jennifer C. Howell, Eugene Chun, Annie N. Farrell, Elizabeth Y. Hur, Courtney M. Caroti, P. Michael Iuvone, Rashidul Haque

**Affiliations:** Department of Ophthalmology, Emory University School of Medicine, Atlanta, GA

## Abstract

**Purpose:**

In recent years, microRNAs (miRNAs) have been reported to play important roles in a broad range of biologic processes, including oxidative stress-mediated ocular diseases. In addition, the polyphenolic compound curcumin has been shown to possess anti-inflammatory, antioxidant, anticancer, antiproliferative, and proapoptotic activities. The aim of this study was to investigate the impact of curcumin on the expression profiles of miRNAs in ARPE-19 cells exposed to oxidative stress.

**Methods:**

MiRNA expression profiles were measured in ARPE-19 cells treated with 20 μΜ curcumin and 200 μΜ H_2_O_2_. PCR array analysis was performed using web-based software from SABiosciences. The cytotoxicity of ARPE-19 cells was determined with the CellTiter-Blue cell viability assay. The effects of curcumin on potential miRNA targets were analyzed with quantitative real-time PCR and western blotting.

**Results:**

Curcumin treatment alone for 6 h had no effect on ARPE-19 cell viability. Incubation with H_2_O_2_ (200 µM) alone for 18 h decreased cell viability by 12.5%. Curcumin alone downregulated 20 miRNAs and upregulated nine miRNAs compared with controls. H_2_O_2_ downregulated 18 miRNAs and upregulated 29 miRNAs. Furthermore, curcumin pretreatment in cells exposed to H_2_O_2_ significantly reduced the H_2_O_2_-induced expression of 17 miRNAs. As determined with quantitative real-time PCR and western blotting, curcumin increased the expression of antioxidant genes and reduced angiotensin II type 1 receptor, nuclear factor-kappa B, and vascular endothelial growth factor expression at the messenger RNA and protein levels.

**Conclusions:**

The results demonstrated that curcumin alters the expression of H_2_O_2_-modulated miRNAs that are putative regulators of antioxidant defense and renin-angiotensin systems, which have been reported to be linked to ocular diseases.

## Introduction

Oxidative stress from reactive oxygen species (ROS) such as hydrogen peroxide (H_2_O_2_) has been implicated in many diseases, including age-related macular degeneration (AMD), in which the retinal pigment epithelium (RPE) is considered the primary target. The RPE is the outermost layer of the retina that absorbs redundant light and processes shed photoreceptor outer segments through phagocytosis, which generates high oxidative stress [1]. Therefore, targeting oxidative damage should be considered as treating and preventing oxidative stress-mediated diseases. Microarray analysis conducted by Weigel et al. [2] and Vandenbroucke et al. [3] revealed that regulation of many genes is altered in cells treated with H_2_O_2_, mediating protective and detrimental cellular effects. Transcriptional regulation in H_2_O_2_-mediated oxidative stress has been shown by many investigators [2-4]. However, the post-transcriptional mechanism of gene expression in response to H_2_O_2_-mediated oxidative stress in RPE cells has not been thoroughly investigated.

Recently, Sun et al. [5] reported microRNA (miRNA/miR) expression profiles were altered by curcumin in pancreatic cancer cells. MiRNA expression profiling of ischemic rat hearts in the context of pretreatments with resveratrol using a quantitative real-time PCR (qRT-PCR)–based assay was conducted by Mukhopadhyay et al. [6]. Curcumin and resveratrol have been shown using oligonucleotide microarray chip and qRT-PCR-based assays to alter the expression profiles of miRNAs in human pancreatic cancer cells and the rat ischemia/reperfusion model, respectively [5,6]. Curcumin significantly protects RPE cells against H_2_O_2_-induced oxidative stress [7]. Baicalein, a naturally occurring flavonoid compound, has also been shown to protect RPE cells against oxidative stress [8].

Curcumin is a naturally occurring phenolic compound derived from the rhizome of *Curcuma longa* and possesses anti-inflammatory and antioxidant effects [9]. Curcumin significantly decreases lipid peroxidation, increases intracellular antioxidant, glutathione, regulates antioxidant enzymes, and scavenges ROS [10,11]. However, the mechanisms underlying the antioxidant activity of curcumin have not been completely delineated. Curcumin has also been studied as a cancer chemopreventive agent in various cancers [12].

In recent years, miRNAs have received greater attention in cancer and other research fields. These small, non-coding RNAs bind to the 3′ untranslated region of target messenger RNA (mRNA) and negatively regulate the expression of genes involved in development, differentiation, proliferation, apoptosis, and other important cellular processes. MiRNAs regulate gene expression at the post-transcriptional level by either degradation or translational repression of a target mRNA. Curcumin regulates the expression of genes involved in regulating cellular signaling pathways, including vascular endothelial growth factor (VEGF), nuclear factor-kappa B (NF-κB), protein kinase B, mitogen-activated protein kinase (MAPK), and other pathways [13], and these signaling pathways could be regulated by miRNAs. In this study, we evaluated the effects of curcumin on protecting RPE cells from H_2_O_2_-induced oxidative stress and identify a potential mechanism. The expression of miRNAs can be measured with northern blot, primer extension assay, RNase protection assay, and global profiling methods [14]. In our investigation, we used a PCR array to profile miRNA expression and to evaluate the effect of curcumin on oxidatively stressed ARPE-19 cells. We hypothesize that curcumin may play an important role in protecting RPE cells from oxidative stress by differentially modulating the expression of miRNAs that putatively regulate the expression of antioxidant, proangiogenic, proliferative, and proinflammatory genes. Our study for the first time reveals that the modulation of miRNA expression may be an important mechanism underlying the biologic effect of curcumin in human RPE, and this approach could be applied as a potential strategy for preventing and treating oxidative stress-mediated ocular diseases such as AMD and diabetic retinopathy (DR).

## Methods

### Cell culture

ARPE-19 cells purchased from American Type Culture Collection (ATCC; Manassas, VA) were cultured at 37 °C in 5% (v/v) of CO_2_ in Dulbecco’s modified Eagle’s medium and Ham’s F12 medium (DMEM/F12) supplemented with 10% fetal bovine serum (Hyclone, Logan, UT), 100 U/ml of penicillin, and 100 µg/ml of streptomycin (Invitrogen, Gibco, Carlsbad, CA). The media were changed every 2–3 days. ARPE-19 cells were seeded in 12-well plates at 1.5×10^5^ cells/well, cultured for 48 h, and then treated with curcumin (Sigma-Aldrich, St. Louis, MO) and H_2_O_2_ (Sigma-Aldrich) alone for 6 h and 18 h, respectively. The effect of curcumin on H_2_O_2_-induced oxidative stress was also assessed, in which ARPE-19 cells were treated with curcumin for 6 h before H_2_O_2_ insult for 18 h and then harvested for miRNA-enriched total RNA or protein extraction. Cells treated with dimethyl sulfoxide (DMSO) were maintained as controls.

### Determination of cell viability

The CellTiter-Blue viability assay (Promega Corp, Madison, WI) was used as the index for cell survival, which measures the ability of living cells to reduce a redox dye (resazurin) into a fluorescent dye (resorufin). The assay was performed according to the manufacturer’s protocol, in which 96-well plates were seeded at 1×10^4^ cells/well and incubated for 6 h for cells to attach to the surface. The ARPE-19 cells were then exposed to varying concentrations (1–50 μM) of curcumin for 6 h. In addition, the cell viability during various durations of exposure of 20 μM curcumin was measured. Cells were washed with phosphate buffered saline (PBS; 10 mM sodium phosphate, 150 mM sodium chloride, pH 7.8), 100 μl of DMEM-F12 without serum was added to each well, and then 20 μl CellTiter-Blue reagent was added. The plates were then incubated at 37 °C for 2 h. The absorbance was recorded at 590 nm in the Synergy 2 Multi-Mode Microplate Reader (Winooski, VT), with the CellTiter-Blue reagent without cells as the blank. The optic density (OD) of the experimental and control samples were subtracted from that of the blank. Cell viability (%) was calculated according to the following formula: Percentage cell viability=(OD of the experimental samples/OD of the control)×100.

### RNA isolation, quantitative real-time polymerase chain reaction, and microRNA polymerase chain reaction arrays

MiRNA-enriched total RNA was extracted from cultured ARPE-19 cells using the QIAzol and miRNeasy kit following the manufacturer’s protocol (Qiagen, Valencia, CA). The concentration of total RNA and the RNA quality (260/280 absorbance ratio) of the samples were measured using a SmartSpec 300 Spectrophotometer (Bio-Rad, Hercules, CA). The first strand kit (Qiagen, cat # 331,401) was used to perform cDNA analysis. For each reaction, 0.8 μg of total RNA, extracted from ARPE-19 cells treated with H_2_O_2_ and with or without curcumin, was submitted to reverse transcription, following the manufacturer’s instructions (Qiagen). The RNA sample with miRNA reverse transcription (RT) enzyme mix was incubated at 37 °C for 2 h, and then the samples were heated at 95 °C for 5 min to degrade RNA and inactivate the reverse transcriptase. To measure miRNAs, the cDNA was diluted tenfold by adding RNase-free H_2_O. The resulting diluted cDNA was added to the RT^2^ Real-Time SYBR Green qPCR Master Mix (Qiagen), which contained real-time PCR buffer, a high-performance HotStart DNA *Taq* polymerase, nucleotides, and SYBR Green dye. The ROX and fluorescein reference dyes were also included in the PCR master mix to normalize variation from well to well.

For PCR array analysis, aliquots of the mixture were placed in each well of a 96-well RT^2^ miRNA profiler miFinder PCR array plate (Qiagen, MAH-001A) that contained a panel of primer sets for a thoroughly researched set of 88 pathway- or disease-focused miRNAs, plus four small nuclear RNA housekeeping (SNORD 44, 47, 48, and U6) assays. The plate also contained duplicate reverse transcription controls that test the efficiency of the RT^2^ miRNA first strand kit (Qiagen) reaction with a primer set detecting the template synthesized from the kit’s built-in miRNA external RNA control and duplicate positive controls that tested the efficiency of the PCR reaction itself using a predispensed artificial DNA sequence and the primer set that detected it. The qRT-PCR analysis was performed in MyiQ Cycler (Bio-Rad Laboratories Inc.) with 25 μl total volume containing diluted cDNA (1 μl per well) and 2X SYBR Green PCR Master Mix. The amplification conditions were the following: 10 min at 95 °C, 40 cycles at 95 °C for 15 s, 60 °C for 30 s, and 72 °C for 30 s. The relative amount of each miRNA in PCR array analysis was normalized to an average of four small nuclear housekeeping genes. Heatmap or cluster analysis was conducted on the expression profiles of all four groups using SABiosciences (Frederick, MD) software.

For mRNA analysis, the isolation of total RNA from ARPE-19 cells and cDNA synthesis were performed using the RNeasy kit and the QuantiTect reverse transcription kit, respectively, according to the manufacturer’s protocol (Qiagen). The qRT-PCR analysis was performed with a 25 μl total volume containing cDNA (2 μl from each sample), 1X QantiFast SYBR Green PCR Master Mix (Qiagen), and 300 nM gene-specific primers (Table 1). The amplification conditions for mRNA qRT-PCR were the following: 5 min at 95 °C, 40 cycles at 95 °C for 10 s, and 60 °C for 30 s. Each sample was assayed in duplicate, and the experimental data were normalized to the expression levels of the housekeeping gene *Hprt.* The absence of non-specific products was confirmed with the analysis of the melt curves and electrophoresis in 2% agarose gels.

**Table 1:** Primers used for quantitative real-time PCR.

Gene	Primer sequence (5’-3’)	Amplicon size (bp)
*VEGF-A*	F: TGCCATCCAATCGAGACCCTG	156
	R:GGTGATGTTGGACTCCTCAGTG	
*NF-κB1*	F:CAACCACAGATGGCACTGCC	125
	R:GCACCAGGTAGTCCACCATG	
*AT_1_R*	F:TGCAGATATTGTGGACACGGCC	154
	R:GTGGGATTTGGCTTTTGGGGG	
*Catalase*	F:CCATTATAAGACTGACCAGGGC	133
	R:AGTCCAGGAGGGGTACTTTCC	
*GPx-1*	F:AGTCGGTGTATGCCTTCTCGG	142
	R:TCGTTCATCTGGGTGTAGTCCC	
*GPx-4*	F:GAGTTTTCCGCCAAGGACATCGA	130
	R:GGTCGACGAGCTGAGTGTAGTTT	
*Hprt*	F:ACAGGACTGAACGTCTTGCTCG	87
	R:TATAGCCCCCCTTGAGCACAC	

FR, fold regulation; FC, fold change. (Note: FC values greater than one indicate a positive or an upregulation, and the FR is equal to the FC. FC values less than one indicate a negative or downregulation, and the FR is the negative inverse of the FC).

The expression levels of mRNA and miRNAs were measured using the threshold cycle (C_t_). The C_t_ is the fractional cycle number at which the fluorescence of each sample passes the fixed threshold. Briefly, the average ΔC_t_ of each group was calculated with the following formula: ΔC_t_=average mRNA/miRNA C_t_ – average of the housekeeping genes C_t_. ΔΔC_t_ was calculated with ΔΔC_t_=ΔC_t_ of the experimental group – ΔC_t_ of the control group. The fold relationships in miRNA or gene expression among the tested samples were calculated using 2^−ΔΔCt^ [15]. The efficiency of reverse transcription in the PCR array was calculated with ΔC_t_=average ΔC_t_^RTC^ – ΔC_t_^PPC^. The ΔC_t_ value of the RT control more than 5 shows evidence of poor reverse transcription efficiency.

### Western blotting analysis

Protein samples were isolated from confluent ARPE-19 cells growing on 12-well plates by washing in ice-cold PBS and then lysed in RIPA buffer (50 mmol/l Tris-HCl [pH 8.0], 150 mmol/l NaCl, 100 μg/ml phenylmethylsulfonyl fluoride, 1% NP-40, 50 mmol/l NaF, 2 mmol/L EDTA), supplemented with protease inhibitor cocktail (Sigma-Aldrich). Samples were centrifuged at 600 × *g* for 30 min at 4 °C to remove cell debris. Protein concentrations were determined with the Lowry method [16]. Samples of 100 μg proteins mixed with loading buffer (Bio-Rad, cat# 161–0791) were boiled for 10 min, separated on 10% Bis–Tris Criterion XT precast gels (Bio-Rad), and transferred onto a polyvinylidene difluoride membrane (Millipore Co., Bedford, MA). Nonspecific binding was blocked by immersing the membrane in 5% dry milk for 3 h. Proteins were incubated with primary antibodies (anti-β-actin, Sigma-Aldrich, 1:3000, cat# A-5441; VEGF-A, Abcam, Cambridge, MA, 1:1000, ab1316; NF-κB, Santa Cruz Biotechnology Inc., Santa Cruz, CA, 1:1000, sc-8008, gift; anti-AT_1_R, Santa Cruz Biotechnology, 1:1000, cat# sc-1173; anticatalase, Santa Cruz Biotechnology, 1:500, cat# sc-34280; anti-GPx-1, Santa Cruz Biotechnology, 1:500, cat# sc-22145; anti-GPx-4, Santa Cruz Biotechnology, 1:500, cat# sc-50497) diluted in 5% bovine serum albumin in PBST (1x PBS, 0.05% Tween-20) overnight at 4 °C. After washing with PBST, the membrane was further incubated with horseradish peroxidase-conjugated antigoat immunoglobulin G (sc-2378, Santa Cruz Biotechnology) at 1:5,000 dilution for 1 h at room temperature. The membrane was washed three times for 15 min each with PBST, and the target proteins were detected with an enhanced chemiluminescence detection system (GE Healthcare, Buckinghamshire, England). The chemiluminescence signal was transferred on Blue Lite Autorad Film (ISC BioExpresss, Kaysville, UT), and the developed film was scanned densitometrically (Kodak Molecular Imaging, Rochester, NY). For data normalization, after the striping procedure, β-actin protein was detected on the same membrane. For β-actin and NF-κB detection, antimouse immunoglobulin G was used as a secondary antibody (sc-2005, Santa Cruz Biotechnology) at 1:5,000 dilution for 1 h at room temperature.

### Statistical analysis

All data including PCR array, qRT-PCR, and immunoblotting analyses were statistically analyzed with Sigma Stat and Sigma Plot (Systat Software, Inc., Chicago IL). Cluster and volcano analyses for the PCR array were done using the PCR array analysis program from SABiosciences. Differences among the groups were analyzed with analysis of variance (ANOVA). Differences between two groups were analyzed with the Student *t* test. In all statistical analyses, p<0.05 was regarded as statistically significant. All values were presented as means±standard error of the mean (SEM).

## Results

### Effect of curcumin on cell viability

**Figure 1:**
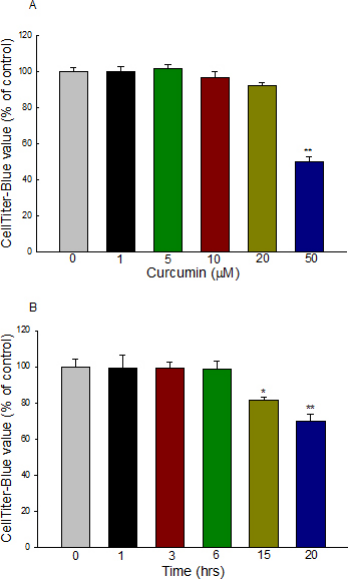
Viability effect of curcumin on ARPE-19 cells at different concentrations and time points. **A**: Dose effect of curcumin on cells treated with different concentrations of curcumin for 6 h. Curcumin at concentrations 1–20 μM did not affect the viability of the ARPE-19 cells. **B**: Time effect of curcumin on cells treated with 20 μM curcumin at different time points. Cells treated with curcumin (20 μM) for 15 h and 20 h induced cell death, compared with the control. Cell survival was determined with CellTiter-Blue assay (absorbance at 560/590 nm). Data represent mean±SEM from five separate samples. *p<0.05 versus control, **p<0.001 versus control.

To determine the effect of curcumin on the cytotoxicity of ARPE-19, cells were grown in 96 wells, and cell viability was assessed with various concentrations (1–50 μM) of curcumin for 6 h or with various durations of exposure to 20 μM curcumin. No significant loss of viability was observed with 1–20 μM curcumin. However, treatment of cells with 50 μM curcumin for 6 h resulted in an approximate 50% decrease in cell viability (p<0.001 versus control). Therefore, 20 μM was chosen as the optimum concentration for subsequent experiments. Curcumin (20 μM) treatment for up to 6 h had no effect on cell viability as measured with CellTiter-Blue, but the CellTiter-Blue value was reduced by 18.5% (p<0.05 versus control) to 30.1% (p<0.001 versus control) of the untreated control cells after 20 µM exposure to 15 and 20 h, respectively (Figure 1). The effect of H_2_O_2_ on the viability of ARPE-19 cells using various concentrations of H_2_O_2_ was previously reported [17]. To examine the effect of H_2_O_2_-mediated oxidative stress in this study, 200 μM H_2_O_2_ was used, as the viability of the cells using this concentration was shown to be only slightly but significantly (p<0.05) reduced by 12.5% compared to control.

### Global microRNA expression profile with polymerase chain reaction array analysis

**Figure 2:**
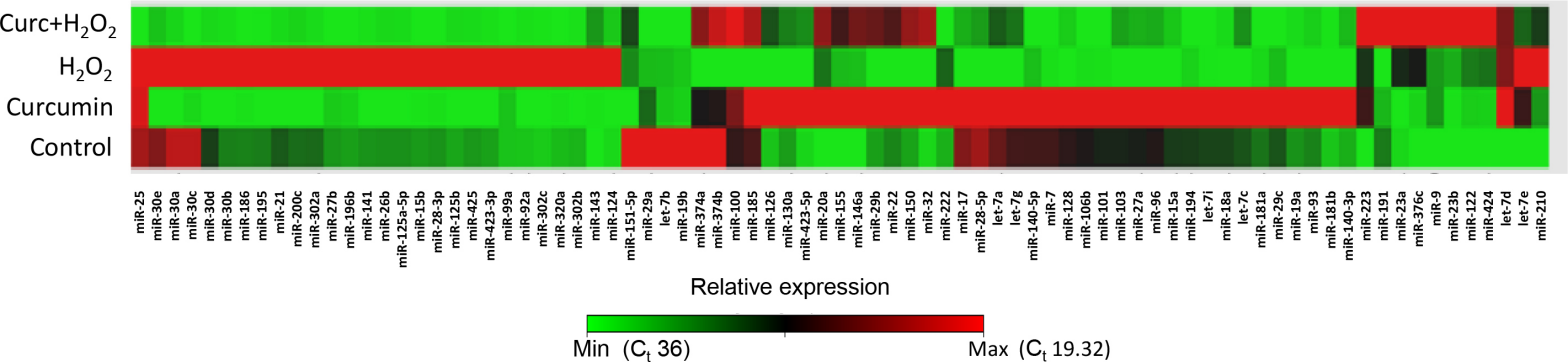
Clustergram or heatmap analysis: Curcumin alters microRNAs (miRNAs) expression profiling in ARPE-19 cells. This clustergram or heatmap represents the expression levels of 81 microRNAs (miRNAs) in four conditions (control, curcumin, hydrogen peroxide [H_2_O_2_], and curc+H_2_O_2_) relative to an average of four housekeeping genes. ARPE-19 cells were treated with 20 μM curcumin and 200 μM H_2_O_2_ for 6 and 18 h, respectively. In the curc+H_2_O_2_ group, the cells were treated with curcumin for 6 h before H_2_O_2_ insult for 18 h. Cells with vehicle DMSO were used as control. Each vertical line in the heatmap represents a single miRNA, and the rows represent groups. Samples clustered according to the condition. Red represents high expression, while green represents low expression. The average C_t_ values for miRNAs with minimum (Min) and Maximum (Max) expression are given in parentheses. Based on clustering, curcumin and H_2_O_2_ alone changed the expression of several miRNAs compared with the control. In addition, curcumin pretreatment altered H_2_O_2_-induced miRNA expression. There were three samples (n=3) in each condition.

To study the responses of miRNAs to curcumin and H_2_O_2_, global miRNA expression profiling using the RT^2^ Profiler miFinder PCR array (SABiosciences) was conducted with miRNA-enriched total RNAs extracted from ARPE-19 cells treated with DMSO (control), curcumin, and H_2_O_2_ in the following ways as shown in Figure 2: 1) DMSO for 6 h; 2) 20 µM curcumin for 6 h; 3) 200 µM H_2_O_2_ for 18 h; and 4) in the curcumin+H_2_O_2_ group, 20 μM curcumin for 6 h before H_2_O_2_ (200 μM) exposure for 18 h. Three experimental assays were performed independently resulting in three PCR array replicates for each condition. In addition, the influence of curcumin was evaluated by comparing the values of the treatment groups with respective non-treated cells exposed to H_2_O_2_ only. Two major bioinformatic approaches were used to analyze the expression of miRNAs and their changes in H_2_O_2_- and curcumin-treated cells.

### Heatmap/clustering analysis

Out of 88 miRNAs surveyed in this array, the qRT-PCR detected 81 miRNAs in ARPE-19 cells. Several observations can be made from Figure 2. In the PCR array, either curcumin or H_2_O_2_ alone altered the expression of several miRNAs that are clustered as red and green, respectively, when compared to the controls. Curcumin and H_2_O_2_ up- or downregulated several miRNAs. Significantly, curcumin pretreatment altered the expression profile of H_2_O_2_-modulated miRNA expression. Five miRNAs (miR-20a, miR-126, miR-146, miR-150, and miR-155) that target VEGF-A, platelet-derived growth factor β (PDGFβ), NF-κB, endothelin 1, p53, and AT_1_R were tightly clustered together in curcumin-treated samples, and their expression was significantly (p<0.05) different compared to the controls. In addition, a tight cluster of nine miRNAs (miR-223, miR-191, miR-23a, miR-376c, miR-9, miR-23b, miR-122, miR-424, and let-7d) was significantly (p<0.05) induced by curcumin compared with the controls. All five members of the miR-30 family (miR-30a-e) were upregulated and downregulated by H_2_O_2_ and curcumin, respectively. However, only miR-30b and miR-30d were differentially expressed more than twofold after H_2_O_2_ treatment.

### Volcano analysis

**Figure 3:**
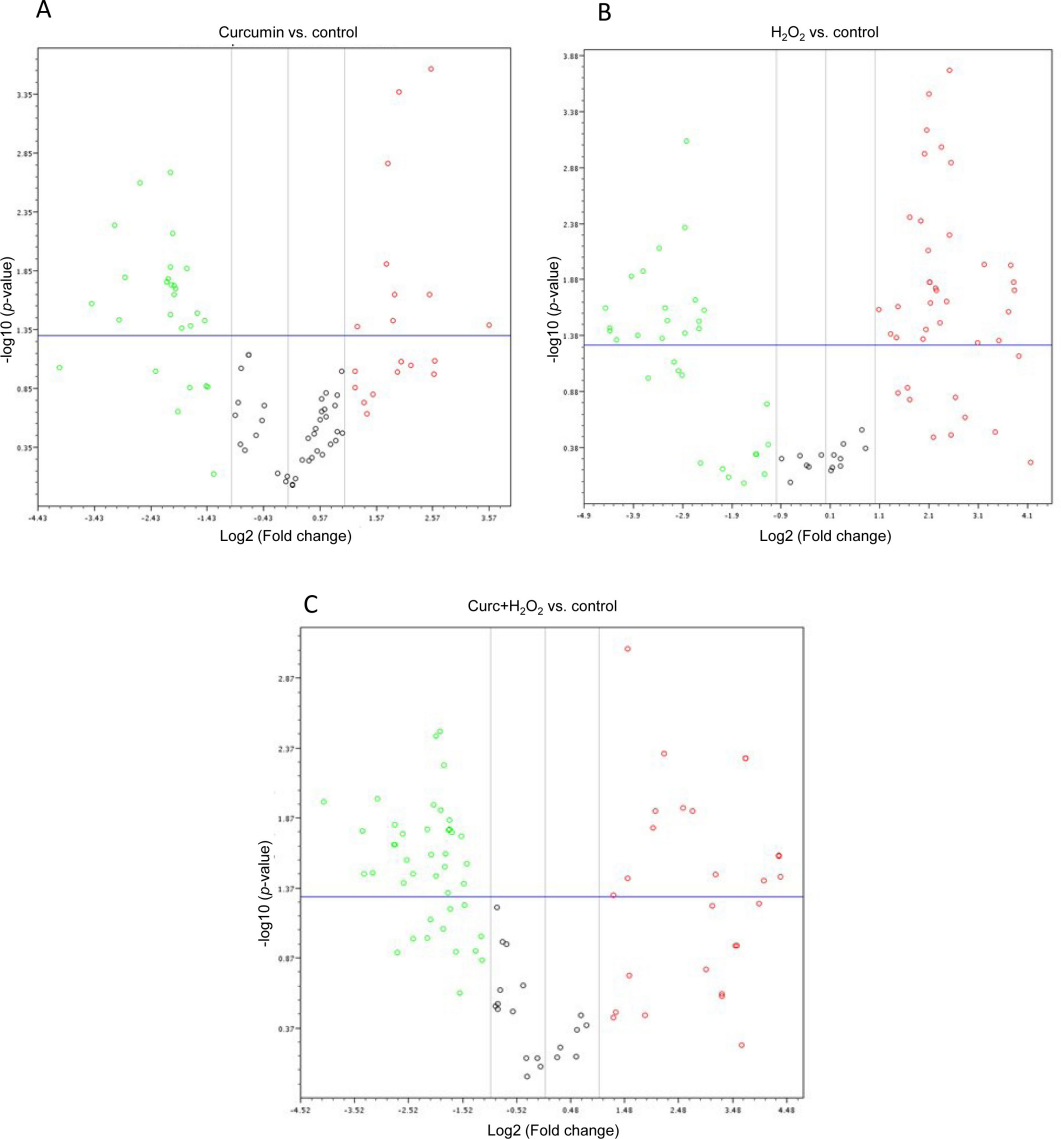
Volcano plot of significance against the relative expression differences between the control and treated groups (**A**–**C**). Each dot represents one of the 81 microRNAs (miRNAs) that was filtered and had detectable expression in either treatment. The *X*-axis displays log2-transformed signal intensity differences between the control group and the experimental group; the Y axis is the log-odds calculated according to the moderated Student t statistic test for differential expression between the control group and the treated group. The horizontal dashed line and the vertical lines represent significance threshold log-odds=2 and twofold expression differences, respectively. All spots above the horizontal dashed line are miRNAs that were identified as showing significant differential expression between the two treatments. MiRNAs positioned in the left and right upper-lateral quadrants represent downregulation and upregulation, respectively.

To compare the miRNA expression levels between two different conditions (curcumin-treated samples to control, H_2_O_2_-treated samples to control, and curcumin+H_2_O_2_-treated samples to control) following the criteria of twofold change in expression and statistical significance (Student *t* test, p<0.05), a volcano plot was generated (Figure 3). The volcano plot also demonstrated that the curcumin pretreatment had altered several H_2_O_2_-modulated miRNAs. In the plot, the first (horizontal) dimension (x-axis) is the fold change (FC) between the two groups (on a log2 scale, so that up- and downregulation appear symmetric), and the second (vertical) axis represents the p value for a Student *t* test of differences between samples (on a negative log scale, so smaller p values appear higher up). The first axis indicates the biologic impact of the change, and the second indicates the statistical evidence, or reliability of the change. For each miRNA, this plot demonstrated the log2 of the FC in the average expression of the two groups (e.g., control versus curcumin-treated samples) as plotted against −log10(p). P represents the probability value for a given miRNA associated with the Student *t* test comparison of the two groups of samples. MiRNAs with statistically significant differential expression were found above the horizontal threshold line of 1.3 (-log of p value=0.05). MiRNAs with 2- or more than 2 FC values would lie to the left (downregulated genes) or right (upregulated genes) of a vertical threshold line. Therefore, significantly upregulated or downregulated miRNAs identified with Student *t* tests would be located in the upper left or upper right parts of the plot. The volcano plot serves as a useful tool for presenting statistically significant results when two groups of samples were compared.

Out of 81 miRNAs screened, based on these criteria (statistical significance and [less than or equal to]-2 or [greater than or equal to] +2 FC values), treating cells with curcumin or H_2_O_2_ alone downregulated 20 (24.69%) and 18 (22.22%) miRNAs, and upregulated nine (11.11%) and 29 (35.8%) miRNAs, respectively, compared to the controls. Pretreatment with curcumin followed by addition of H_2_O_2_ down- and upregulated 29 (35.8%) and 14 (17.28%) miRNAs, respectively, when compared to controls. Out of 29 miRNAs downregulated by curcumin pretreatment, 17 miRNAs were induced by H_2_O_2_ alone based on two criteria, i.e., statistical significance and 2-FC. The volcano analysis for the effect of curcumin on miRNA expression has been arranged into two categories.

### Curcumin-downregulated microRNAs

**Figure 4:**
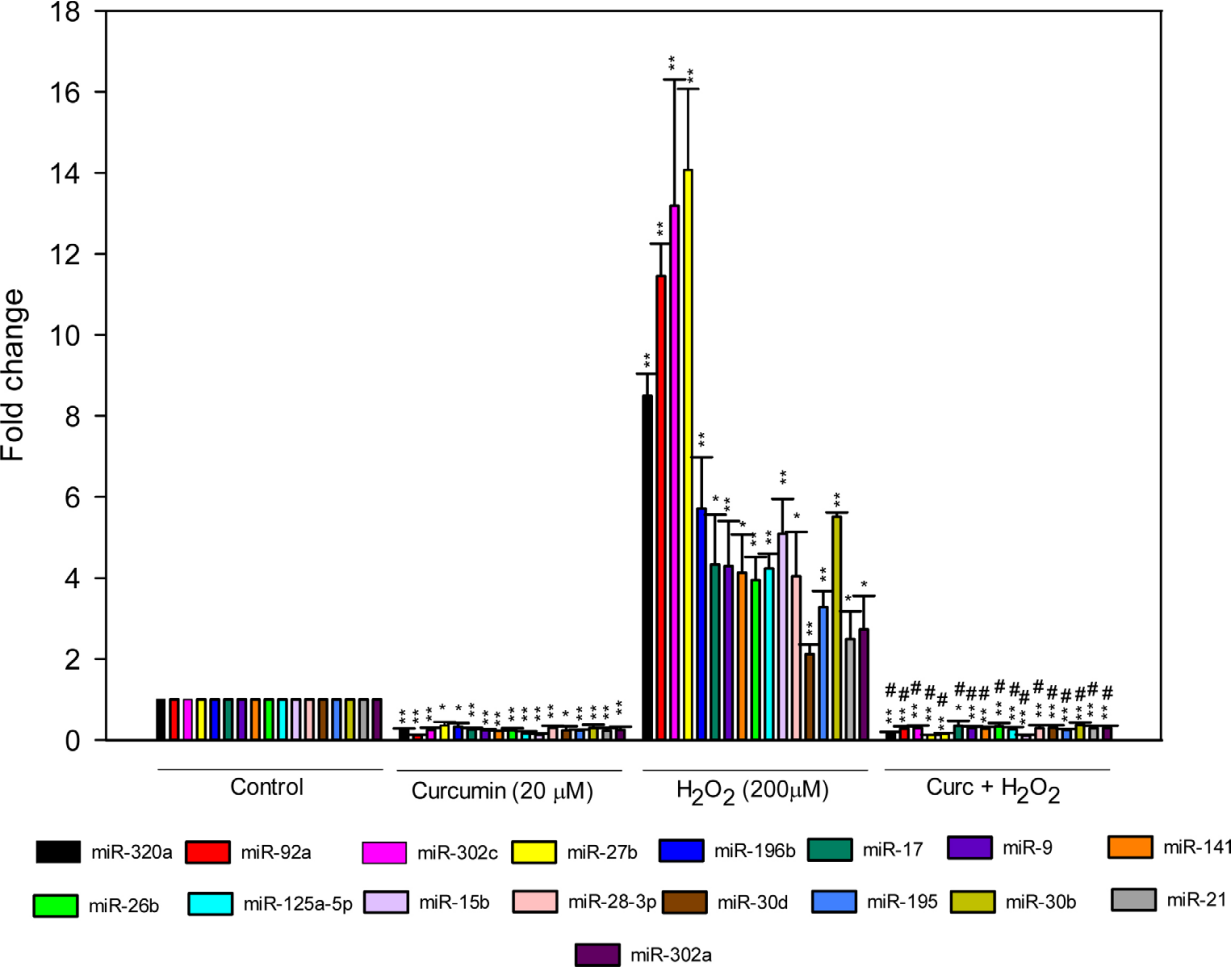
Curcumin attenuated hydrogen peroxide-induced microRNA expression in ARPE-19 cells. In the polymerase chain reaction (PCR) array, cells were treated with 20 μM curcumin for 6 h and 200 μM hydrogen peroxide (H_2_O_2_) for 18 h. In the curcumin pretreatment group, the cells were treated with 20 μM curcumin for 6 h first, and after washing, the cells were insulted with 200 μM H_2_O_2_ for 18 h. The curcumin pretreatment significantly suppressed the H_2_O_2_-induced expression of 17 microRNAs (miRNAs) compared to controls. Data represent mean±SEM from three separate samples. *p<0.05 versus control, **p<0.001 versus control, #p<0.001 versus H_2_O_2_, FC≥ or ≤2.

**Figure 5:**
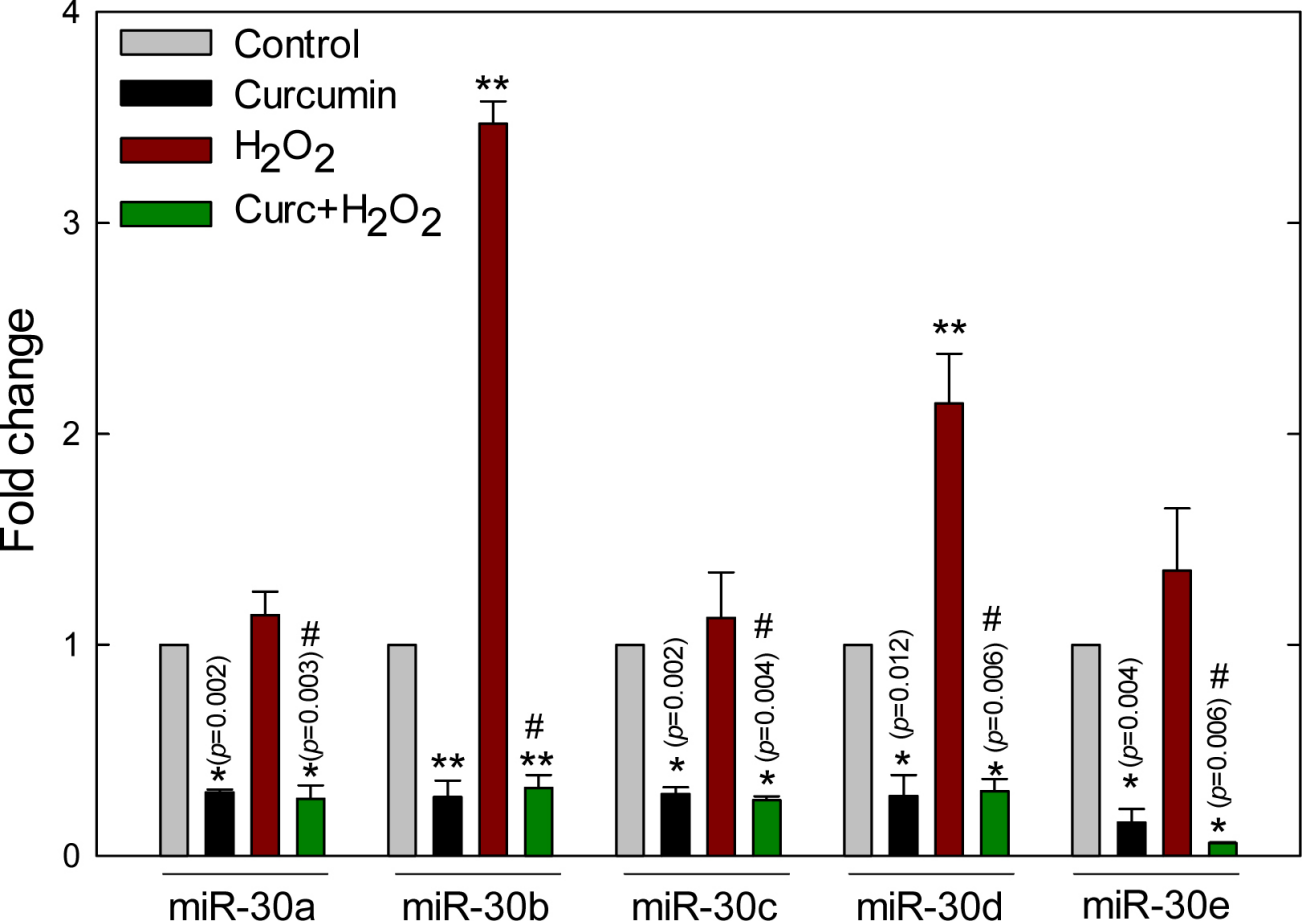
Curcumin alters hydrogen peroxide-induced miR-30 expression in ARPE-19 cells. As measured with the quantitative reverse-transcription polymerase chain reaction (qRT-PCR)–based array, cells treated with 200 μM hydrogen peroxide (H_2_O_2_) for 18 h significantly induced miR-30b and miR-30d expression, compared to controls. However, curcumin and curcumin pretreatments for 6 h significantly reduced all five members of miR-30, when compared with controls. In the curcumin pretreated group, the cells were treated with curcumin for 6 h first, and then the cells were incubated for 18 h with H2O2. Data represent mean±SEM from three separate samples. *p<0.05 versus control, **p<0.001 versus control, #p<0.001 versus H_2_O_2_, FC≥ or ≤2.

Based on statistical significance (p<0.05) and 2-FC, curcumin pretreatment attenuated the H_2_O_2_-induced expression of 17 miRNAs (miR-15b, miR-17, miR-21, miR-26b, miR-27b, miR-28–3p, miR-30b, miR-30d, miR-92a, miR-125a-5p, miR-141, miR-196b,, miR-195, miR-302a, miR-302c, miR-320a, and miR-9), which were also significantly reduced by the curcumin treatment alone (Figure 4, Table 2). Out of 17 H_2_O_2_-induced miRNAs, the maximum miRNA expression induced by H_2_O_2_ was observed for miR-124 (14.23 FC, p=0.017), and the most downregulated miRNA expression by curcumin pretreatment was observed for miR-30e (−17.16 FC, p=0.014). In the array, miR-23b, another member of the miR-23–27–24 clusters, was also significantly up- and downregulated by H_2_O_2_ and curcumin, respectively. Two members of the miR-30 family (miR-30b and miR-30d), putative regulators of the genes implicated in oxidative stress-mediated ocular diseases, were significantly (miR-30b: p=0.001, FC 3.52; miR-30d: p=0.001, FC 2.14) upregulated under the oxidative environment, and curcumin alone significantly (p<0.05) reduced the expression of all five members of the miR-30 family, compared to the controls, and significantly reduced induction by H_2_O_2_ (Figure 5).

**Table 2:** List of miRNAs altered by curcumin and H_2_O_2_.

Control versus curcumin					Control versus H_2_O_2_			Control versus H_2_O_2_+curcumin				
**Downregulated**					**Downregulated**			**Downregulated**				
miRNA (miR)		P value		FR (FC)	miRNA (miR)	P value	FR (FC)	miRNA (miR)		P value		FR (FC)
miR-302c		0.033		−4.26 (0.24)	miR-15a	0.041	−13.98 (0.07)	miR-7		0.011		−4.18 (0.24)
miR-30e		0.016		−7.37 (0.14)	let-7a	0.031	−5.91 (0.17)	miR-30d		0.014		−3.40 (0.29)
miR-30d		0.019		−4.19 (0.24)	miR-126	0.045	−19.01 (0.05)	miR-140–5p		0.017		−10.42 (0.10)
miR-30b		0.02		−3.97 (0.30)	miR-27a	0.024	−22.15 (0.05)	miR-141		0.031		−3.62 (0.28)
miR-141		0.017		−4.46 (0.22)	miR-29a	0.036	−20.81 (0.05)	miR-92a		0.025		−3.60 (0.28)
miR-92a		0.006		−8.39 (0.12)	miR-29b	0.038	−20.86 (0.05)	miR-26b		0.018		−2.94 (0.34)
miR-27b		0.037		−2.78 (0.36)	miR-22	0.024	−9.57 (0.10)	miR-196b		0.015		−6.84 (0.15)
miR-30a		0.007		−3.33 (0.30)	Let-7d	0.001	−7.09 (0.14)	miR-30a		0.006		−3.67 (0.27)
miR-26b		0.013		−4.26 (0.24)	let-7i	0.036	−9.51 (0.17)	miR-30b		0.029		−2.72 (0.37)
miR-17		0.023		−4.06 (0.25)	miR-106b	0.04	−7.22 (0.14)	miR-423–3p		0.04		−6.15 (0.16)
miR-15b		0.037		−7.94 (0.13)	let-7g	0.005	−7.23 (0.14)	let-7i		0.027		−5.91 (0.17)
miR-302a		0.019		−4.06 (0.25)	miR-28–5p	0.011	−12.99 (0.08)	miR-106b		0.034		−9.15 (0.11)
miR-30c		0.014		−3.46 (0.29)	miR-374a	0.044	−9.93 (0.10)	miR-17		0.04		−2.84 (0.35)
miR-9		0.007		−4.13 (0.24)	miR-100	0.007	−10.4 (0.10)	miR-15b		0.034		−10.18 (0.10)
miR-196b		0.032		−3.05 (0.33)	miR-103	0.02	−6.27 (0.16)	miR-302a		0.017		−3.30 (0.30)
miR-125a-5p		0.003		−6.16 (0.16)	miR-128	0.012	−15.4 (0.06)	miR-29c		0.021		−6.95 (0.14)
miR-195		0.002		−4.26 (0.24)	miR-150	0.031	−9.29 (0.11)	miR-186		0.016		−4.51 (0.22)
miR-423–3p		0.027		−11.23(0.09)	miR-7c	0.024	−5.52 (0.18)	miR-21		0.017		−3.45 (0.29)
miR-21		0.017		−4.35 (0.23)	**Upregulated**			miR-302c		0.047		−3.46 (0.29)
miR-28–3p		0.041		−3.30 (0.30)	miR-30d	0.001	2.14	miR-9		0.016		−3.39 (0.30)
**Upregulated**					miR-23a	0.014	4.32	miR-30c		0.012		−3.80 (0.26)
miR-18a	0.042		2.35		miR-142–3p	0.021	4.36	miR-320a		0.034		−5.42 (0.18)
miR-142–3p	0.022		3.71		miR-143	0.037	10.83	miR-125a-5p		0.003		−3.84 (0.26)
miR-146a	0.037		3.64		miR-124	0.017	14.23	miR-195		0.004		−4.06 (0.25)
miR-20a	0.002		3.4		miR-122	0.004	3.82	miR-28–3p		0.036		−4.06 (0.25)
miR-126	0.041		11.9		miR-125b	0.032	5	miR-128		0.018		−6.17 (0.16)
miR-29b	0.001		5.79		miR-141	0.001	4.13	miR-27b		0.009		−8.62 (0.12)
miR-22	0.001		3.91		miR-9	0.001	4.28	miR-30e		0.014		−17.16 (0.06)
miR-150	0.022		5.7		miR-92a	0.046	11.45	miR-143		0.021		−6.89 (0.15)
miR-155	0.012		3.33		miR-27b	0.014	14.06	**Upregulated**				
					miR-26b	0.044	3.95	miR-22	0.012		20.1	
					miR-96	0.016	4.69	let-7b	0.025		20.1	
					miR-21	0.04	2.49	miR-424	0.035		8.93	
					miR-23b	0.009	9.34	miR-99a	0.012		6.66	
					miR-17	0.014	4.33	let-7d	0.038		16.63	
					miR-15b	0.001	5.09	miR-150	0.005		4.59	
					miR-302a	0.043	2.73	miR-20a	0.001		2.89	
					miR-186	0.037	4.11	miR-23b	0.011		5.89	
					miR-223	0.017	4.76	miR-29a	0.012		4.12	
					miR-320a	0.048	8.46	miR-29b	0.016		4.02	
					miR-196b	0.005	5.71	miR-155	0.049		2.4	
					miR-302b	0.001	5.85	miR-146a	0.037		2.89	
					miR-125a-5p	0.007	4.23	miR-126	0.006		4.35	
					miR-195	0.004	3.28	miR-142–3p	0.036		20.57	
					miR-28–3p	0.001	4.04					
					miR-302c	0.026	13.19					
					miR-423–3p	0.001	5.71					
					miR-30b	0.001	3.52					
					miR-30d	0.024	2.12					

### Curcumin-induced microRNAs

**Figure 6:**
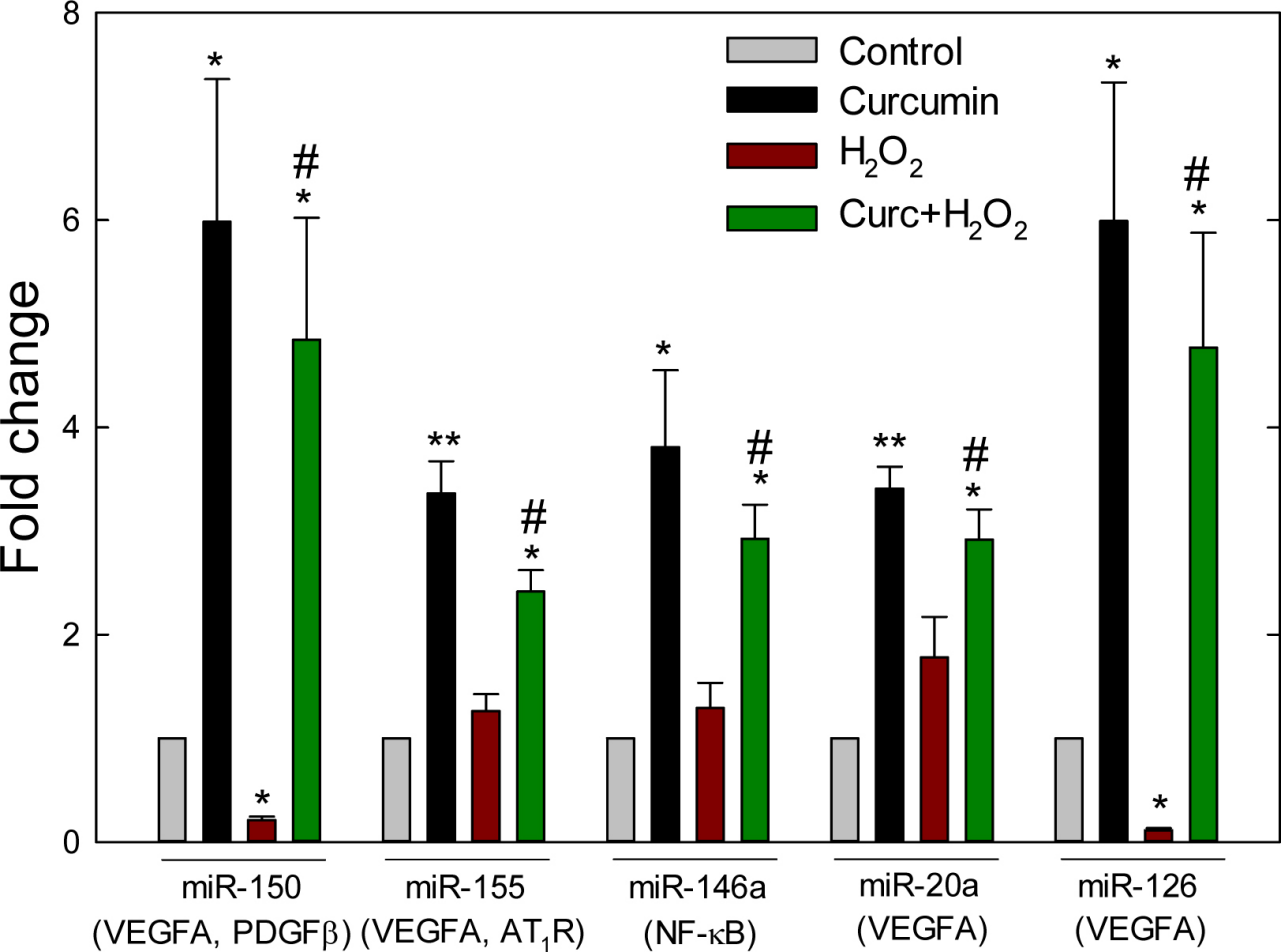
Curcumin-induced microRNAs in ARPE-19 cells. Following two criteria of statistical significance (p<0.05) and fold change (FC) (≥ or ≤2), the quantitative real-time polymerase chain reaction (qRT-PCR) array revealed five microRNAs (miRNAs) that were upregulated in cells treated with 20 μM curcumin for 6 h. The miRNAs have been reported to target angiotensin II type 1 receptor (AT1R), nuclear factor-kappa B (NF-κB), platelet-derived growth factor β (PDGFβ), and vascular endothelial growth factor (VEGF). Data represent mean±SEM from three separate samples. Data represent mean±SEM from three separate samples. *p<0.05 versus control, **p<0.001 versus control, #p<0.05 versus H2O2.

Based on statistical significance (p<0.05) and 2-FC, curcumin alone significantly increased the expression of nine miRNAs (miR-18a, miR-22, miR-20a, miR-29b, miR-126, miR-142–3p, miR-146a, miR-150, and miR-155). However, compared to controls, curcumin pretreatment upregulated 14 H_2_O_2_-modulated miRNAs of which seven miRNAs (miR-20a: p=0.001, FC=2.89; miR-126: p=0.006, FC=4.35; miR-146a: p=0.037, FC=2.89; miR-150: p=0.005, FC=4.59; miR-155: p=0.049, FC=2.40; miR-29b: p=0.016, FC=4.02; and miR-142–3p: p=0.036, FC=20.57) were also significantly upregulated by curcumin treatment alone. Five miRNAs (miR-150: p=0.031, FC=−9.29; miR-126: p=0.045, FC=−19.01; miR-29a: p=0.036, FC=−20.81; miR-29b: p=0.038, FC=−20.86; let-7d: p=0.001, FC=−7.09) out of 14 miRNA induced by curcumin pretreatment were significantly downregulated by H_2_O_2_ treatment (Figure 6, Table 2).

In addition to two major groups, PCR revealed a third group in which miRNAs were downregulated by H_2_O_2_ and curcumin. Out of 81 miRNAs examined and followed by two criteria (statistical significance and 2-FC), the expression of three H_2_O_2_-downregulated miRNAs (let-7i, miR-106b, and miR-128) was significantly downregulated by curcumin pretreatment.

### Effect of curcumin on gene expression

**Figure 7:**
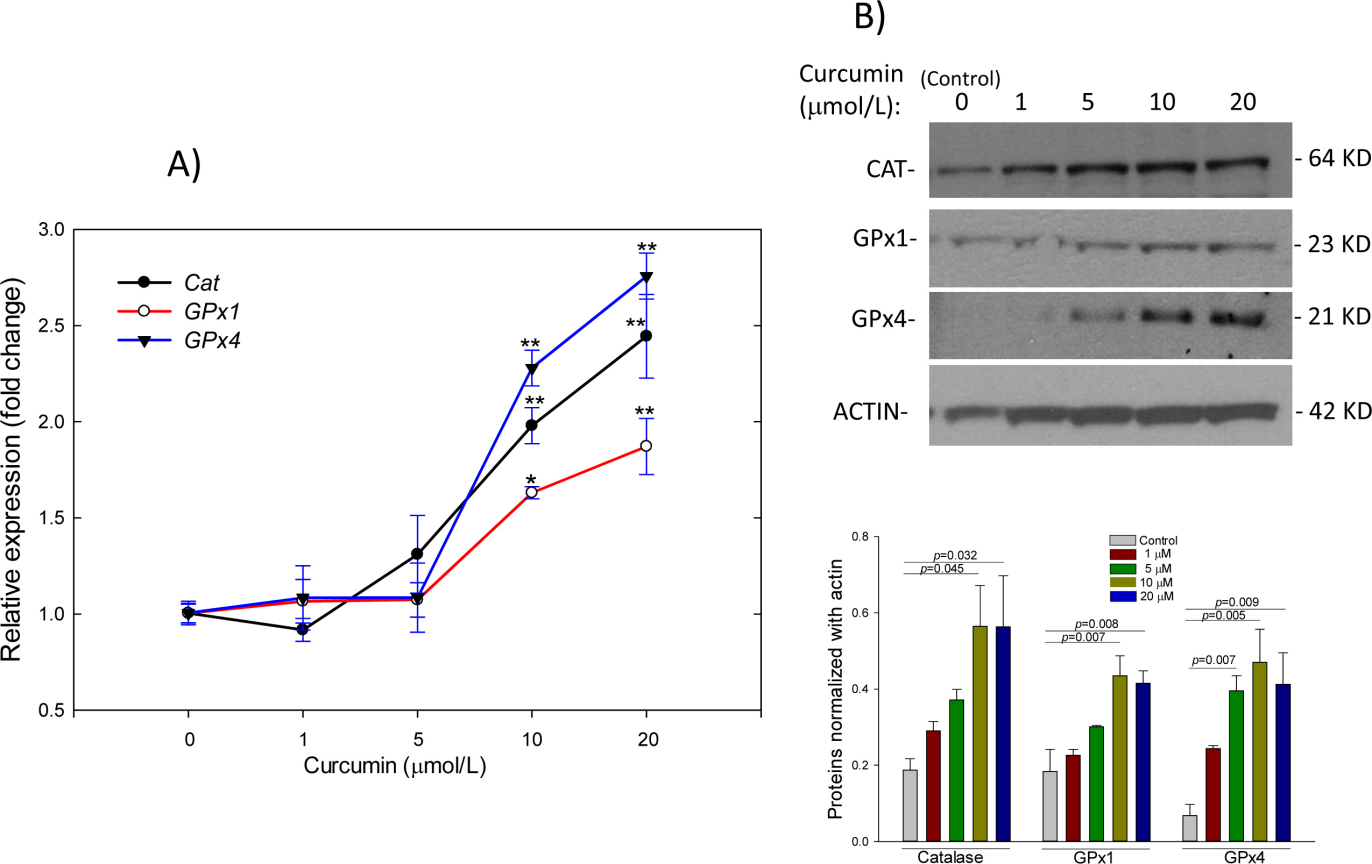
Dose effect of curcumin on catalase and Gpx-s messenger RNA (**A**) and protein (**B**) levels in ARPE-19 cells. Cells treated with various concentrations of curcumin (1–20 μΜ) for 6 h dose-dependently increased catalase, GPx-1, and GPx-4 at the messenger RNA (mRNA) and protein levels, compared with controls. Representative western blots of catalase (CAT), GPx-1, GPx-4, actin, and densitometric analysis of protein levels are shown in the upper and lower panels, respectively. Data represent mean±SEM from four separate samples. *p<0.05 versus control, **p<0.001 versus control.

**Figure 8:**
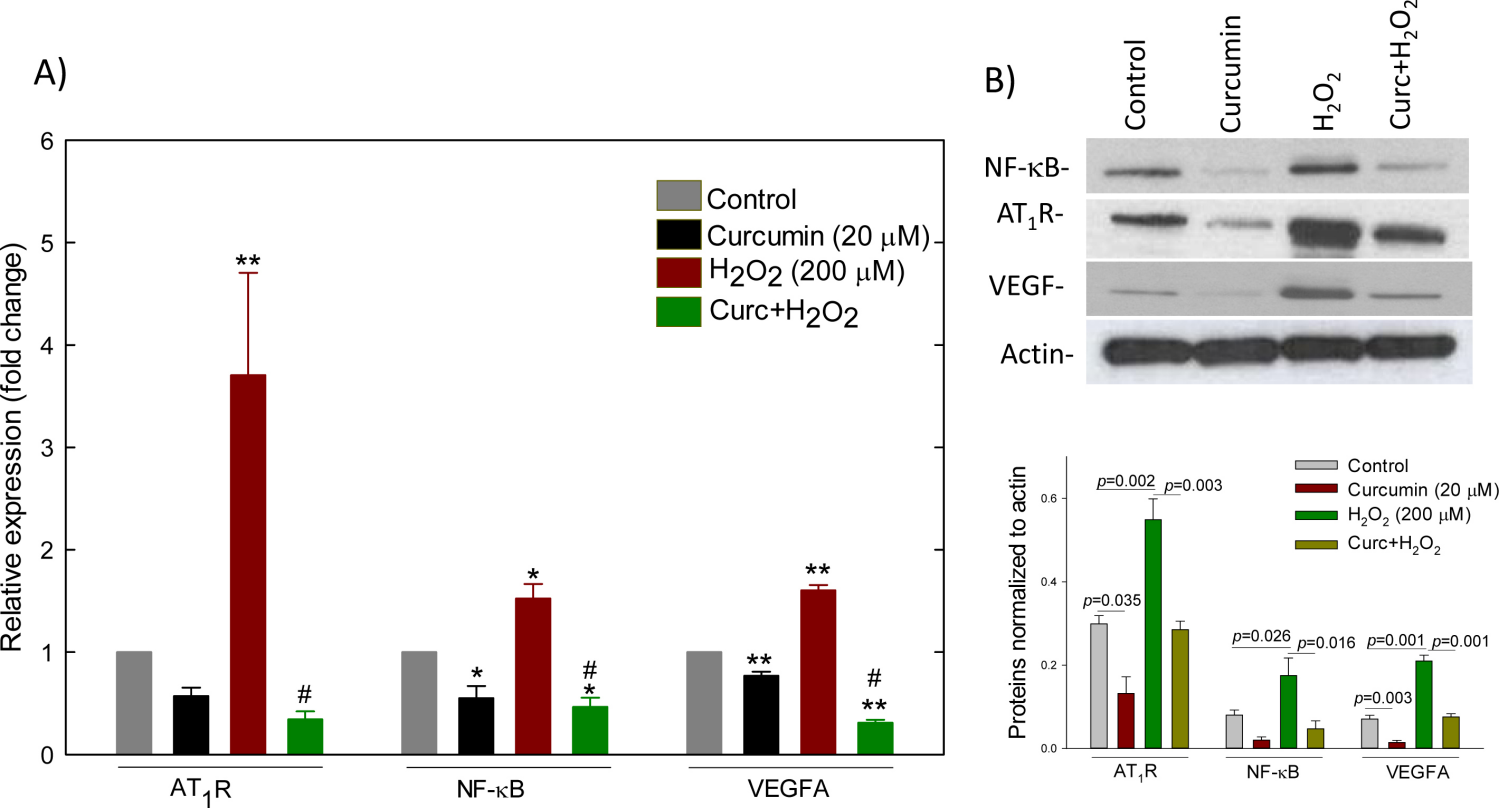
Curcumin attenuated hydrogen peroxide (H_2_O_2_)-induced expression of angiotensin II type 1 receptor, nuclear factor-kappa B, and vascular endothelial growth factor at the mRNA (**A**) and protein (**B**) levels in ARPE-19 cells. The mRNA and protein measurements were done with quantitative real-time polymerase chain reaction (qRT-PCR) and western blotting, respectively. Cells were treated with curcumin and hydrogen peroxide (H_2_O_2_) for 6 and 18 h, respectively. In the curc+H_2_O_2_ group, cells were treated with 6 h followed by insult with 200 μM H_2_O_2_ for 18 h; cells were washed with fresh media in-between the curcumin and H_2_O_2_ treatments. Representative western blots of angiotensin II type 1 receptor (AT_1_R), nuclear factor-kappa B (NF-κB), and actin, and densitometric analysis of the protein levels are shown in the upper and lower panels, respectively (**B**). Data represent mean±SEM from three (for western) or four (for qRT-PCR) separate samples. *p<0.05 versus control, **p<0.001 versus control, #p<0.001 versus H_2_O_2_.

We evaluated the effect of curcumin on the expression of catalase, GPx-s, AT_1_R, NF-κB, and VEGF-A at the mRNA and protein levels. Exposure of ARPE-19 cells to various concentrations of curcumin (1–20 μM) for 6 h resulted in a concentration-dependent increase in catalase and GPx-s expression at the mRNA and protein levels (Figure 7). The increase in catalase and GPx-s expression at concentrations of 10 μM and above was significantly different from the vehicle (ethanol)-treated cells (control, p<0.001). Compared with the control, a sublethal concentration of H_2_O_2_ (200 μM) significantly induced (p<0.001) the expression of AT_1_R, NF-κB, and VEGF-A at the mRNA and protein levels (Figure 8). Curcumin (20 μM) significantly reduced the expression of NF-κB (p<0.05) and VEGF-A (p<0.001) at the mRNA and protein levels. In addition, curcumin pretreatment significantly (p<0.001) attenuated the H_2_O_2_-induced expression of AT_1_R, NF-κB, and VEGF-A at the mRNA and protein levels (Figure 8), indicating that the activation of AT_1_R, NF-κB, and VEGF-A is mediated by a prooxidant mechanism.

## Discussion

This study explored the potential modulation of miRNAs by curcumin in ARPE-19 cells using PCR array and identified several H_2_O_2_-modulated miRNAs whose expression was altered by curcumin. Dietary polyphenolic components such as curcumin have been implicated in many biologic pathways involved in development, differentiation, apoptosis, proliferation, and cellular stress signaling [9,18,19]. These processes have been reported to be regulated by miRNAs [20-22]. Bioinformatic analysis showed that a single miRNA is capable of modulating the expression of more than 100 mRNA targets and more than 50% of human protein coding genes could be regulated by miRNAs [23]. Therefore, to investigate the functional aspects of miRNAs, array-based miRNA surveys and other high-throughput approaches are becoming increasingly popular in biologic sciences. To date, 1,527 human mature miRNAs have been reported (miRBase 18). However, the exact number of ocular miRNAs expressed in the human retina or RPE is not yet known. For the first time, we report the effect of curcumin on the expression profiles of miRNAs in ARPE-19 cells, a cellular model for human retinal pigment epithelium.

Curcumin or H_2_O_2_ treatment significantly affected the levels of many miRNAs in ARPE-19 cells. In general, more miRNAs were upregulated than downregulated in response to H_2_O_2_ treatment, while curcumin treatment primarily downregulated expression. Of the miRNAs that were affected by both treatments, the direction (up- or downregulation) was opposite in all cases except one, miR-142–3p. In addition, curcumin counteracted the upregulation of miR expression by H_2_O_2_ treatment.

H_2_O_2_ treatment significantly upregulated miR-30b and miR-30d, two members of the miR-30 family, which is consistent with our previous results [17], and all five members of the family were downregulated by the curcumin treatments. The mechanism of ROS-mediated gene regulation of miR-30b and miR-30d seems to be different from that of the other three members of the family. In silico analysis suggests that the expression of miR-30b and miR-30d, but not the other three members of the family, is regulated by the promoter of the zinc finger and AT hook domain–containing (*ZFAT*) gene. The epigenetic [2] and transcription factor-mediated regulation [3] of miRNA genes may underlie the molecular mechanism of ROS-mediated regulation of miRNAs in ARPE-19 cells.

Curcumin and other dietary components have been reported to alter the expression profiles of miRNAs in other tissues. In the human pancreatic cell line, curcumin has been shown to significantly up- and downregulate 11 and 18 miRNAs, respectively [5]. Consistent with those data, in our study, curcumin upregulated miR-103, miR-22, and miR-23b and downregulated miR-195, miR-15b, miR-196, and miR-92. In the human multidrug-resistant adenocarcinoma cell line A549/DDP, curcumin altered miRNA expression and significantly downregulated the expression of miR-186 [24], a negative regulator of the proapoptotic purinergic P2X7 receptor [25], which is also consistent with our result.

Curcumin was shown to significantly downregulate the H_2_O_2_-induced expression of miR-302 cluster in ARPE-19 cells. MiR-302 has been reported to inhibit several epigenetic regulators, including AOF1/2, methyl-CpG binding proteins 1 and 2, and DNA (cytosine-5-)-methyltransferase 1, that induce global DNA demethylation and subsequently activate transcription factors Oct4, Sox2, and Nanog [26]. The treatment of ARPE-19 cells with H_2_O_2_ in our experiment induced miR-26b, miR-15b, and miR-9, and that induction was significantly suppressed by curcumin. The oxidant-induced expression of these three miRNAs showed consistency with the result shown in ARPE-19 cells treated with a retinoic acid derivative (4HPR), which induces ROS generation [27]. MiR-21 has been shown to protect cardiac myocytes against H_2_O_2_-induced injury via targeting the programmed cell death protein 4 and activator protein-1 pathway [28]. Our data also showed that miR-21 was sensitive to H_2_O_2_ stimulation, and expression of miR-21 was significantly downregulated by curcumin pretreatment. The miR-17–92 cluster is expressed in human retinoblastoma, and upon deletion of *Rb* family members, miR-17–92 overexpression leads to explosive development of retinoblastoma [29]. In our investigation, miR-17 and miR-92 of the cluster were induced by H_2_O_2_-mediated stress, whereas curcumin treatments significantly downregulated the expression of the cluster.

The actions of curcumin and resveratrol, a structurally-related polyphenolic compound, are similar in some respects [30,31]. Curcumin and resveratrol target many of the same signaling molecules, including NF-κB, B-cell lymphoma 2, B-cell lymphoma-extra large, Bim, and survivin [32]. Curcumin and resveratrol have antioxidant effects that protect ARPE-19 cells from cytotoxicity [7,33,34]. Although the effects of resveratrol on miRNA expression in RPE cells have not been reported to our knowledge, resveratrol affects miRNA expression in several other cell types, and miRNA regulation is increasingly thought of as a means of delivering the beneficial effects of resveratrol [35]

A major challenge for retinal miRNA studies is identifying relevant genes and their downstream targets that regulate angiogenesis and increased vascular permeability, the major factors for wet AMD and DR [36]. AMD and DR are the leading causes of blindness. Oxidative stress-mediated increases of VEGF, vascular endothelial growth factor receptor, Ang II, AT_1_R, NF-κB, and transforming growth factor beta promote angiogenesis and increased vascular permeability; these are the well-recognized regulatory factors for wet AMD and proliferative DR. Our qRT-PCR and immunoblotting data showed that the expression of AT_1_R, VEGF, and NF-κB was strongly upregulated with H_2_O_2_-mediated oxidative stress at the mRNA and protein levels. However, curcumin not only reversed the H_2_O_2_-mediated expression but also significantly decreased their expression compared with control. In our investigation, curcumin significantly induced the expression of five miRNAs (miR-146a, miR-150, miR-155, miR-20a, miR-22, and miR-126) that target downstream molecules such as VEGF, NF-κB, PDGFβ, and endothelin 1. The mechanism of action of curcumin on the modulation of miRNA expression is not well understood. The altered expression of miRNAs can occur via several molecular mechanisms such as transcriptional regulation, post-transcriptional processing, genomic abnormalities [37], and regulation by epigenetic factors [38]. MiR-146a was shown to be transactivated by NF-κB, but also to inhibit NF-κB activation, showing negative feedback regulation on NF-κB activation [39]. VEGF was reported to induce miR-20a and miR-155 in human umbilical vein endothelial cells [40]. However, miR-20a was also reported to target VEGF, indicating negative feedback regulation of miR-20a on VEGF [41]. MiR-150 and miR-155 were also reported to regulate PDGFβ [42] and AT_1_R [43], respectively. The miR-23–27–24 clusters enhance angiogenesis and choroidal neovascularization in mice by repressing sprouty2 and Sema6a proteins, which negatively regulate MAPK and VEGFR2 signaling in response to angiogenic factors [44]. In our analysis, curcumin downregulated two members of the clusters, miR-23b and miR-27b, which were upregulated by H_2_O_2_-mediated oxidative stress.

Curcumin has been known to target several biochemical and molecular signaling cascades either through direct binding to proteins or through modulation of gene expression [45]. Curcumin has been shown to physically interact with 33 proteins and is known to modulate various molecular targets, including cytokines, transcription factors, growth factors and their receptors, and genes controlling cell proliferation and apoptosis [46]. The inhibitory effect of curcumin on carcinogenic, angiogenic, inflammatory, and proliferative properties is mediated through suppression of a host of cell-signaling molecules, including activator protein-1 [46], NF-κB [46], early growth response-1 factor [47], tumor necrosis factor alpha [48,49], cytokines [49], IκB kinase phosphorylation [50], Janus kinase-2 phosphorylation [51], c-Jun N-terminal kinase [52], phosphoinositide 3-kinase/protein kinase B phosphorylation [53,54] and p38 MAPK [54,55], extracellular signal-regulated kinase phosphorylation [56], epidermal growth factor receptor [54], tyrosine kinase, serine/threonine and tyrosine protein kinase [46,56], matrix metalloprotease-9 [57], nitric oxide synthase [55], PDGF and epidermal growth factor [58], transforming growth factor beta-1, connective tissue growth factor, VEGF, and vascular endothelial growth factor receptor [56,59,60]. Curcumin-mediated reduction of NF-κB protein in our experiments could be through direct interaction with the protein, as curcumin was shown to directly bind to NF-κB and the binding was reversed by glutathione [61].

In summary, we evaluated the effect of curcumin on the expression levels of miRNA in ARPE-19 cells in the presence of an oxidative environment. For the first time, we have shown that this polyphenolic compound can alter the expression profiles of H_2_O_2_-modulated miRNAs in this human RPE culture system. Modulation of miRNA expression may be an important mechanism in the pathogenesis of AMD and DR, and curcumin may provide a therapeutic approach for preventing and treating these oxidative stress-mediated diseases.
